# Femtosecond X-ray cross-correlation analysis of disordered crystals forming in a supercooled atomic liquid

**DOI:** 10.1107/S2052252525004063

**Published:** 2025-06-03

**Authors:** Johannes Möller, Michele Caresana, Alexander Schottelius, Felix Lehmkühler, Ulrike Boesenberg, Frédéric Caupin, Francesco Dallari, Tiberio A. Ezquerra, José M. Fernández, Luca Gelisio, Claudia Goy, Jörg Hallmann, Anton Kalinin, Chan Kim, Ruslan P. Kurta, Dmitry Lapkin, Francesco Mambretti, Markus Scholz, Roman Shayduk, René Steinbrügge, Florian Trinter, Ivan A. Vartanyants, Alexey Zozulya, Davide E. Galli, Gerhard Grübel, Anders Madsen, Robert E. Grisenti

**Affiliations:** a European X-Ray Free-Electron Laser Facility, 22869Schenefeld, Germany; b Institut für Kernphysik, J. W. Goethe-Universität Frankfurt am Main, 60438Frankfurt am Main, Germany; c Deutsches Elektronen-Synchrotron DESY 22607Hamburg Germany; d The Hamburg Centre for Ultrafast Imaging, 22761Hamburg, Germany; e Institut Lumière Matière, Université Claude Bernard Lyon 1, CNRS, Institut Universitaire de France,69622Villeurbanne France; f Macromolecular Physics Department, Instituto de Estructura de la Materia, IEM-CSIC, 28006Madrid, Spain; g Laboratory of Molecular Fluid Dynamics, Instituto de Estructura de la Materia, IEM-CSIC, 28006Madrid, Spain; h GSI Helmholtzzentrum für Schwerionenforschung GmbH, 64291Darmstadt, Germany; i Dipartimento di Fisica, Università degli Studi di Milano, 20133Milano, Italy; j Molecular Physics Fritz-Haber-Institut der Max-Planck-Gesellschaft,14195Berlin Germany; Brookhaven National Laboratory, USA

**Keywords:** femtosecond studies, nanocrystals, correlated fluctuations, diffract-then-destroy, dynamical studies, time-resolved studies, X-ray free-electron lasers, XFELs

## Abstract

We demonstrate an advanced scattering method for accessing the 3D reciprocal space of crystalline structures forming in a rapidly supercooled noble-gas liquid using a combination of femtosecond X-ray diffraction and X-ray cross-correlation analysis. The preservation of angular information from the scattering signal allows probing the structure factor along selected directions in reciprocal space and identifying signatures undetectable in azimuthally integrated scattering curves. Therefore, more information from serial diffraction experiments on stochastic crystallization processes can be retrieved despite the inherent variation of the crystal orientation and morphology for each single probe. We also demonstrate how different features in the correlation maps can be associated with certain forms of stacking faults, which enables studying such defects *in situ* and disentangling them from simultaneous changes in crystal size and temperature.

## Abstract 2

We demonstrate an advanced scattering method for accessing the 3D reciprocal space of crystalline structures forming in a rapidly supercooled noble-gas liquid using a combination of femtosecond X-ray diffraction and X-ray cross-correlation analysis.

## 1. Introduction

Crystallization, the transition from a disordered liquid or melt into a long-range ordered crystalline structure, is a fundamental process with significant implications in both basic and applied research. However, studying crystallization at the atomic or molecular level presents major experimental challenges because of its spontaneous nature, as well as the extremely short time- and small length-scales involved. The advent of X-ray free-electron lasers (XFELs) has opened new avenues for investigating these rapid processes, offering the ability to probe matter with unprecedented spatial and temporal resolution.

Crystallization proceeds through the spontaneous, thermally induced formation of a critical nucleus of the new ordered phase, a process commonly described in the framework of classical nucleation theory (CNT) (Kelton & Greer, 2010 ▸; Karthika *et al.*, 2016 ▸). The critical seed subsequently grows into a stable macroscopic crystalline structure. However, many systems, including colloids and protein solutions (Wolde & Frenkel, 1997 ▸; Vekilov, 2004 ▸; Sanz *et al.*, 2007 ▸; Sauter *et al.*, 2015 ▸) as well as atomic-scale materials (Chung *et al.*, 2009 ▸), have been observed to follow more complex crystallization pathways. For example, according to Ostwald’s rule of stages (Ostwald, 1897 ▸), intermediate metastable polymorphs with lower activation barriers may form before the final crystal structure emerges. To fully understand crystallization mechanisms, it is therefore essential to provide insights into structural features along the crystallization pathway, beginning possibly with the earliest nucleation events.

For a wide range of materials, crystallographic defects play a crucial role in determining mechanical, electrical, and microstructural properties (Koren *et al.*, 2014 ▸; Su *et al.*, 2021 ▸; An *et al.*, 2019 ▸; Lähnemann *et al.*, 2014 ▸; Li *et al.*, 2010 ▸). These defects influence properties such as material strength, ductility, and conductivity, making their characterization vital for optimizing material performance. In systems with face-centered cubic (FCC) or hexagonal close-packed (HCP) structures, the small energy difference between these phases frequently leads to stacking faults (Hull & Bacon, 2011 ▸; Kühnel *et al.*, 2011 ▸). Such defects arise when the ideal FCC stacking sequence (*ABCABC*…) is disrupted, either by skipping a layer (*ABABC*…) or by inverting the stacking direction (*ABCBAC*…, twinning). The stacking fault probabilities, denoted α and β, respectively, describe deviations from the ideal FCC (α = β = 0) or HCP (α = β = 1) structures, with α = β = 0.5 representing a random HCP (rHCP) configuration.

Experimentally, stacking faults are often studied using techniques such as transmission electron microscopy or scanning tunneling microscopy (Li *et al.*, 2010 ▸; Shih *et al.*, 2021 ▸; Gutierrez-Urrutia *et al.*, 2010 ▸), which typically only provide *ex situ* and relatively localized information. It is also difficult to extend these techniques to statistical investigations, in particular to study rapid crystallization as a stochastic process *in situ*. While X-ray diffraction (XRD) enables *in situ* probing on large ensembles, conventional methods typically yield orientationally averaged data, for instance by analysis of line profiles in powder diffraction averages (Warren, 1969 ▸; Velterop *et al.*, 2000 ▸; Kim *et al.*, 2023 ▸; Fletcher *et al.*, 2024 ▸). This can hinder the distinction between different crystalline defects especially at low densities and limit the ability to resolve statistically relevant structural features.

In a recent study, we performed femtosecond XRD experiments on supercooled atomic liquids of argon and krypton using the European X-ray free-electron laser (EuXFEL) (Möller *et al.*, 2024 ▸). Our measurements revealed that crystal nucleation rates were lower by factors of 100–1000 compared with predictions from CNT. Recent computer simulations of water have shown that stacking-disordered crystallites can be more stable than hexagonal ice, leading to higher nucleation rates than those predicted by CNT (Lupi *et al.*, 2017 ▸). These findings suggest that crystal nucleation is intimately linked to the structural morphology of the emerging crystalline phase. Understanding this connection requires detailed insight into the early stage morphological features of growing crystals in a supercooled liquid, thus highlighting the need for an experimental approach capable of resolving these structural characteristics. In this work, using the same dataset as Möller *et al.* (2024 ▸), we take a step toward addressing this challenge. We demonstrate that the unique beam properties of EuXFEL – including high peak flux, femtosecond pulse duration, and large number of pulses per second – combined with X-ray cross-correlation analysis (XCCA) enable the detailed probing of reciprocal space during the crystallization process.

XCCA (Wochner *et al.*, 2009 ▸; Altarelli *et al.*, 2010 ▸; Kirian, 2012 ▸), also known as fluctuation X-ray scattering (Martin, 2017 ▸; Martin *et al.*, 2020 ▸) or correlated X-ray scattering (Mendez *et al.*, 2014 ▸; Mendez *et al.*, 2016 ▸), probes angular correlations in scattered X-ray patterns in order to determine the structure of single scattering entities (Kam, 1977 ▸; Saldin *et al.*, 2010 ▸; Saldin *et al.*, 2011 ▸; Kurta *et al.*, 2017 ▸; Pande *et al.*, 2018 ▸); or to obtain local order, arrangements, or orientation (Martin, 2017 ▸; Martin *et al.*, 2020 ▸; Lehmkühler *et al.*, 2016 ▸; Lehmkühler *et al.*, 2018 ▸; Kurta *et al.*, 2019 ▸; Zaluzhnyy *et al.*, 2017 ▸; Zaluzhnyy *et al.*, 2019 ▸; Schulz *et al.*, 2020 ▸; Niozu *et al.*, 2021 ▸; Lapkin *et al.*, 2022 ▸). Previous studies on crystalline features and defects were performed on nanometre-length scales investigating colloidal samples (Wochner *et al.*, 2009 ▸; Lapkin *et al.*, 2022 ▸; Lehmkühler *et al.*, 2016 ▸) or have been restricted to a limited number of scattering vectors (Mendez *et al.*, 2016 ▸; Niozu *et al.*, 2020 ▸), thus providing limited reciprocal space coverage. Here, we present the first *in situ* study applying XCCA to crystallization at atomic length scales, with sufficient statistical accuracy to probe the entire, 3D reciprocal space. One can therefore retrieve structural information from specific directions in reciprocal space containing features defined by crystalline defects and also follow their evolution during the experiment.

## 2. Experimental

**Figure 1:**
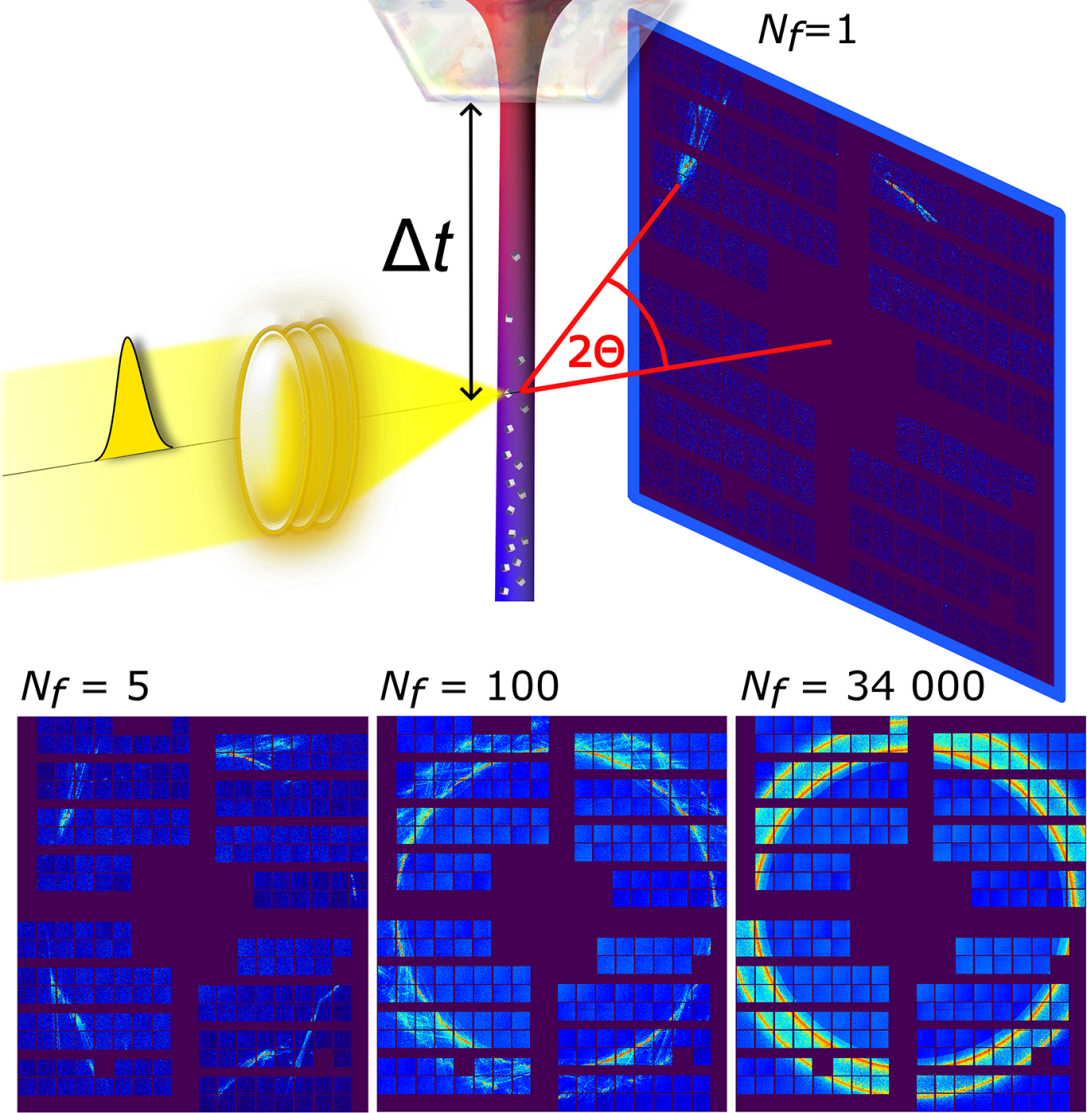
Sketch of the experimental setup. A liquid jet of krypton (3.5 µm diameter, 76 m s^−1^ velocity, 122 K exit temperature at the nozzle tip) was injected into a vacuum and subject to evaporative cooling. The formation of crystals was probed via femtosecond X-ray pulses (<100 fs, λ = 0.128 nm) focused down to 300 nm × 300 nm size. Scattering acquisitions were taken as a function of distance to the nozzle orifice, which translated to different times Δ*t* after the onset of rapid cooling. Examples of scattering images (maximum per pixel) from *N_f_* = 5, 100, and 34000 single acquisitions at Δ*t* > 8.5 µs are displayed in the bottom row.

The experiments were performed at the Materials Imaging and Dynamics (MID) instrument of EuXFEL (Möller *et al.*, 2024 ▸; Madsen *et al.*, 2021 ▸; Decking *et al.*, 2020 ▸), with supporting experiments at the P01 beamline of PETRA III (Wille *et al.*, 2010 ▸). A temperature-controlled jet of liquid krypton was injected into vacuum, where it rapidly cooled to well below its melting point (115.8 K) by surface evaporation, so spontaneous crystallization could be observed with the onset of homogeneous nucleation. Jet freezing was probed as a function of the distance *z* from the nozzle, which is equivalent to considering different times Δ*t* = *z*/*v* (Schottelius *et al.*, 2020 ▸; Möller *et al.*, 2024 ▸), by virtue of the jet propagation velocity *v*. The distance *z* as well as the stability of the jet were monitored using a side-view microscope with a resolution of <1 µm. We restrict our analysis to a range of Δ*t* where a high hit rate Φ > 0.5 was obtained, as detailed in the supporting information. An extension to even earlier moments of the crystallization process is foreseen for future studies. At MID, about 3500 pulse trains, each with 50 X-ray pulses, were recorded for every probed nozzle distance using the Adaptive Gain Integrating Pixel Detector (AGIPD) (Allahgholi *et al.*, 2019 ▸; Sztuk-Dambietz *et al.*, 2023 ▸). This resulted in 80 000 to 160 000 usable scattering patterns per Δ*t* point, recorded within 6 min each. Due to the high repetition rate of EuXFEL, the entire data set for this publication was recorded in 70 min, easing challenges to stable liquid-jet operation while allowing systematic measurements with a large number of statistically independent scattering patterns. A sketch of the experimental setup is displayed in Fig. 1 ▸, with *q* = 4π/λsin(θ) being the wavevector transfer. In order to remove the partly dominating scattering contribution from the liquid phase, we developed a data-reduction routine, which uses the burst-mode acquisition enabled by the EuXFEL pulse structure [details are provided in the supporting information and in Möller *et al.* (2024 ▸)]. The reduced scattering intensity is denoted 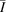 in the following.

**Figure 2:**
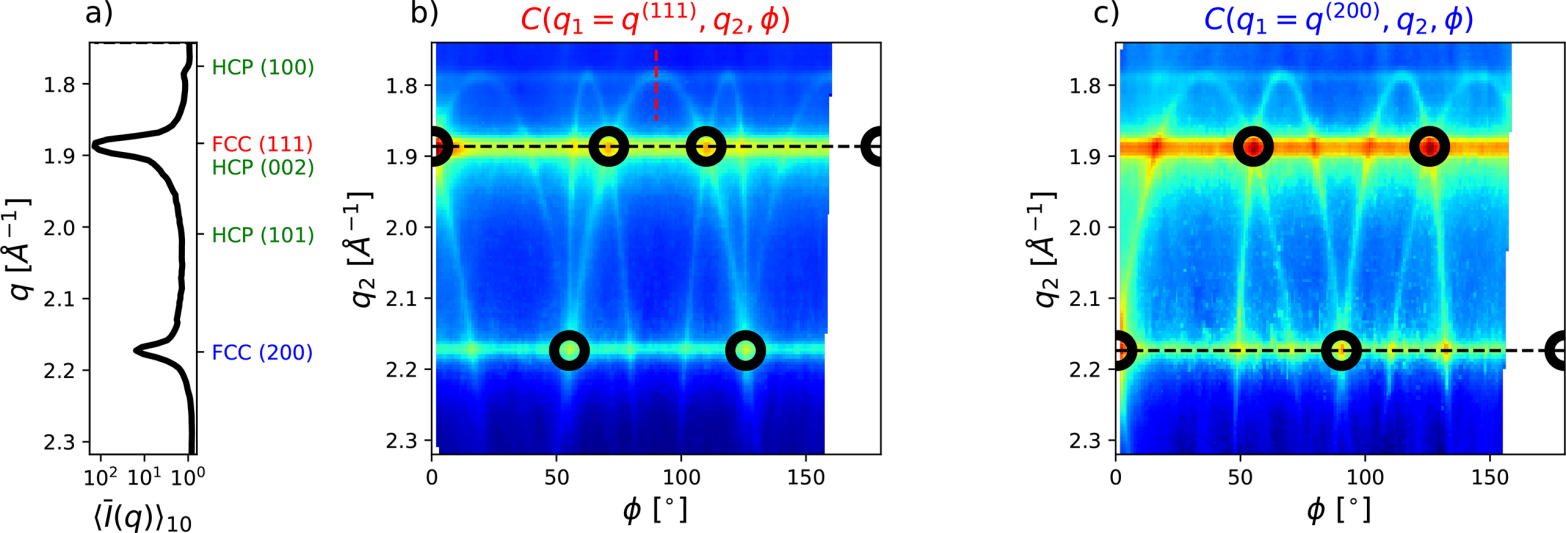
(*a*) Azimuthal integration of an averaged, background-subtracted detector signal. Locations of FCC and HCP lattice reflections are marked. (*b*) and (*c*) XCCA maps *C*(*q*_1_, *q*_2_, ϕ) measured at a distance to the nozzle of 598 µm, corresponding to Δ*t* = 7.8 µs. (*b*) is sliced at the FCC (111) peak position (*q*_1_ = *q*^(111)^) and (*c*) at the FCC (200) position. Black dashed lines mark *q*_1_ = *q*_2_ correlations and black circles mark the expected peak positions assuming a perfect FCC crystal. The red dashed line in panel (*b*) marks the slice at 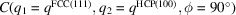, as further discussed in Section 4.

A single, background-subtracted scattering acquisition is shown in Fig. 1 ▸, with the occurrence of elongated streaks rather than symmetrical, well defined Bragg spots on the detector. Increasing the number of acquisitions (*N_f_* = 5, 100, and 34000), it becomes evident that the frequently appearing streaks primarily connect the (111) and (200) reflections of an FCC lattice, already identified as the most prominent reflections (Möller *et al.*, 2024 ▸; Schottelius *et al.*, 2020 ▸), as also displayed in Fig. 2 ▸(*a*). Similar streaks have been observed in X-ray scattering from systems that are subject to stacking faults, for example, for ice (Esmaeildoost *et al.*, 2022 ▸) or solid Xe clusters (Niozu *et al.*, 2021 ▸). In the latter case, the analysis was performed by selecting specific single-scattering acquisitions having a favorable orientation, from which the stacking sequence of three individual crystals could be obtained. The analyses demonstrated a coexistence of FCC and rHCP domains for three selected crystallites, but no conclusions could be drawn about the probabilities of such stacking faults in the full ensemble. In Niozu *et al.* (2020 ▸), it was also shown that such crystalline defects can give rise to specific features in angular correlations of selected iso-*q* rings (rings of constant |**q**|) on the detector. Due to the high repetition rate of EuXFEL, which allows femtosecond X-ray scattering experiments with a high number of acquisitions, we can extend this approach to access the entire 3D correlation space, only limited by the largest *q* accessible in the experiment. This becomes possible since the high frame rate allows the collection of many measurements of all crystal orientations, so that a well defined average of the full correlation space can be probed. Thus, a deeper insight into the nature of the ensemble’s stacking faults during the crystallization process can be obtained.

## 3. X-ray cross-correlation analysis

The XCCA correlation function is expressed as the sum of pixel pairs within each (*q*_1_, *q*_2_, ϕ) bin, weighted by the scattering intensity product of both detector pixels,
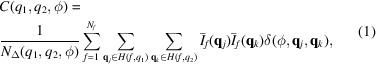
where 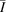 is the background-reduced scattering intensity (see Section S2 of the supporting information); *N_f_* is the number of recorded frames; *H*(*f*, *q*) is the set of lit pixels in each *q* bin and frame *f*, defined as *H*(*f*, *q*) = 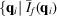 > 10 arb.u. and 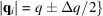; and 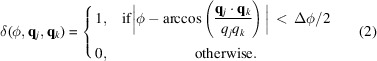
The number of pixel pairs on the detector for each combination of (*q*_1_, *q*_2_, ϕ) is given by 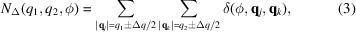
where Δ*q* = 0.005 Å^−1^ and Δϕ = 1°. Specific slices are shown for two selected *q*_1_ values in Figs. 2 ▸(*b*) and 2 ▸(*c*). An animated view of the full correlation map can be also found in the supporting information. The dashed black lines mark the *C*(*q*_1_ = *q*_2_, ϕ) cuts for the most intense reflections of *q*^(111)^ and *q*^(200)^, which were accessible in previous FEL studies (Mendez *et al.*, 2014 ▸; Mendez *et al.*, 2016 ▸; Niozu *et al.*, 2021 ▸) and also in storage-ring data from beamline P01 obtained by us (Fig. S8 of the supporting information). Strong, angle-independent contributions are visible at the location of the two main reflections, which can stem from multiple grains or crystallites with random orientation to each other. The subtraction of this contribution is described in Section S5 of the supporting information. For a perfect FCC crystal, well separated peaks would only appear for angles between the respective lattice vectors in reciprocal space, marked by black circles here, *e.g.* at ϕ = 70.5° and ϕ = 109.5° [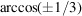] for *q*_1_ = *q*_2_ = *q*^(111)^ and at ϕ = 90° for *q*_1_ = *q*_2_ = *q*^(200)^. The presence of additional peaks already indicates the occurrence of some form of crystal defects, but no conclusion about the nature of these defects can be drawn from the data along the black dashed lines alone.

### 3.1. Correlations in reciprocal space

**Figure 3:**
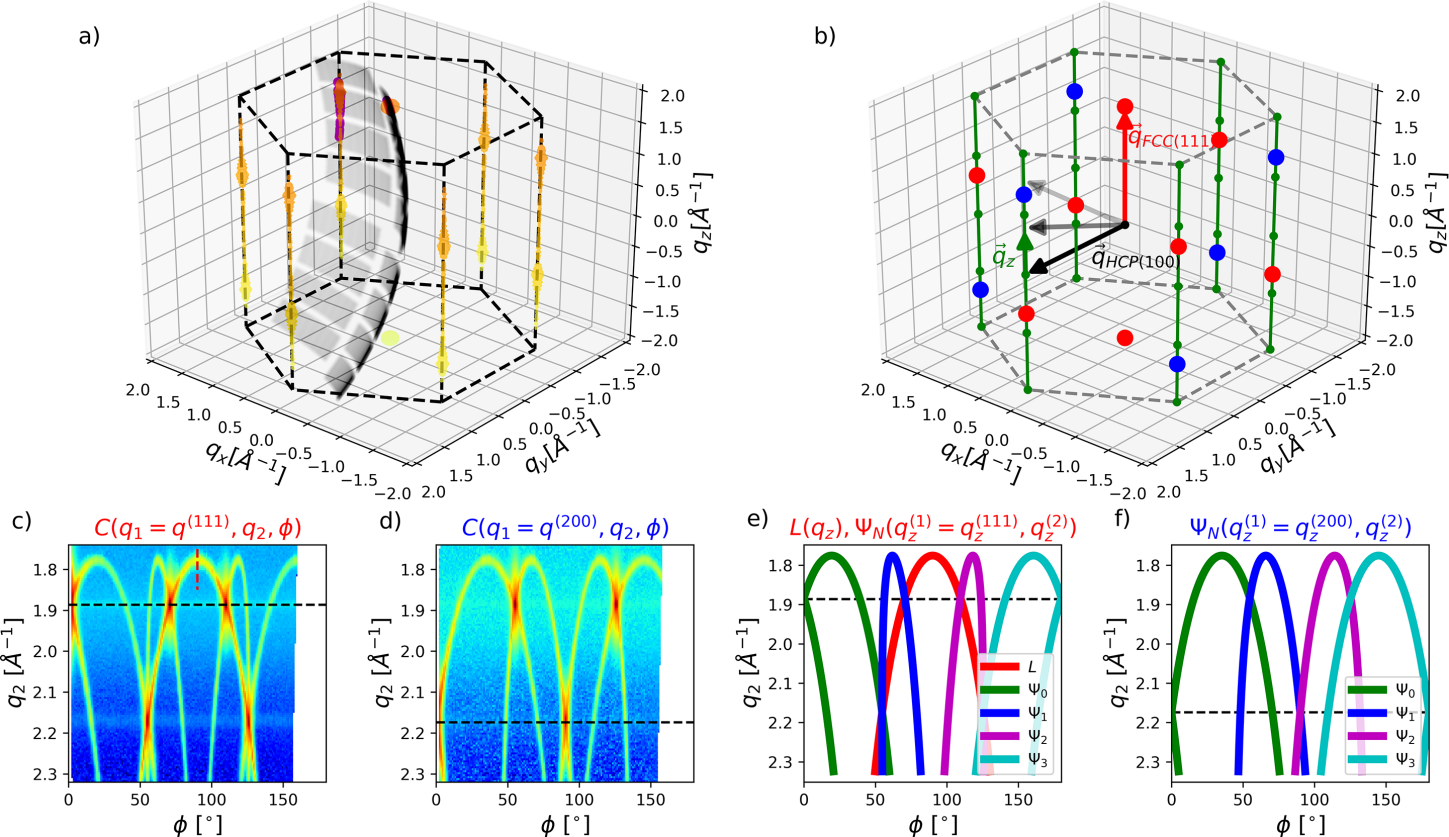
(*a*) 3D representation of one simulated particle’s *S*(*q_x_*, *q_y_*, *q_z_*). Black dashed lines are plotted as a guide to the eye. The randomly oriented slice of the reciprocal space by the Ewald sphere is depicted as a gray surface, which is only shown for the actual positions of detector pixels on the surface. Positions where the detector slices *S*(*q_x_*, *q_y_*, *q_z_*) are shown in magenta. (*b*) Reciprocal lattice points of HCP (green), FCC (111) (red) and (200) (blue). The reciprocal lattice vectors of one FCC (111) (red arrow) and one HCP (100) (black arrow) are additionally depicted. In order to parameterize the lines and surface in the (*q*_1_, *q*_2_, ϕ) correlation space, the vector **q**_*z*_ = **q**_2_ − **q**_HCP(100)_ along a rod (green line) is introduced. (*c*) and (*d*) Simulated XCCA maps for the same *q* values as in Fig. 2 ▸ and α = 0.05 stacking fault probability. (*e*) and (*f*) Slices through the surfaces in correlation space Ψ, given by the correlation of different rods emerging from stacking faults. The parametrization is detailed in equations (S3) and (S4) of the supporting information.

The full, 3D correlation maps in Figs. 2 ▸(*b*) and 2 ▸(*c*) show more complex parabola-like line features, which connect the main FCC reflections. These stem from straight features like Bragg rods in reciprocal space, which manifest themselves as curved signals in the correlation space (*q*_1_, *q*_2_, ϕ). Below we confirm by both numerical calculations and geometrical considerations – which are in fairly good agreement with the experimental data – that these features originate from stacking faults in the crystals. The numerical calculations are detailed in Section S3 of the supporting information. Fig. 3 ▸(*a*) depicts the resulting modulus squared structure factor *S*(*q_x_*, *q_y_*, *q_z_*) (proportional to the scattered intensity) computed for a 50 nm sized crystallite by assuming a stacking fault probability α = 0.05. Elongated features in the *q_z_* direction can be observed, from which the exact stacking sequence of a single crystal could potentially be retrieved (Lapkin *et al.*, 2022 ▸; Niozu *et al.*, 2020 ▸; Niozu *et al.*, 2021 ▸). Due to the random orientation of each probed crystal, the curvature of the Ewald sphere and the finite active area of the detector, this information is, however, not fully accessible in the experiment. This is illustrated by a gray surface, which shows the probed part of reciprocal space by one scattering acquisition with random crystal orientation. The next acquisition in the experiment would probe a different crystal with a different orientation.

By calculating XCCA maps from many such randomly oriented scattering acquisitions, a more detailed understanding of the reciprocal space can still be obtained. We show this by calculating *N*_*f*_ = 7.5 × 10^5^ independent scattering images with random orientation from ten different particles’ *S*(*q_x_*, *q_y_*, *q_z_*), each generated with α = 0.05 but different actual stacking sequences. From these, the correlation maps are calculated the same way as for the experimental data and displayed in Figs. 3 ▸(*c*) and 3 ▸(*d*).

The same parabola-like correlation features can be observed as previously shown in Figs. 2 ▸(*b*) and 2 ▸(*c*). In fact, each line in the correlation maps can be explained by the correlations originating from the vertical rods in reciprocal space, as further illustrated in Fig. 3 ▸(*b*). Each vertical rod (green line) consists of one FCC (111) (red) and one FCC (200) (blue) lattice point, as well as several HCP lattice points (green). Correlations can occur either between a rod (black vectors) and a single FCC (111) lattice point (red vector) at *q_x_* = *q_y_* = 0 Å^−1^ [*L*(*q*_*z*_), equation (S3)] or between two rods. In the following, the coordinate in reciprocal space along the direction of a rod will be denoted by *q_z_*, defined by **q**_*z*_ = **q**_2_ − **q**_HCP(100)_ in accordance with Niozu *et al.* (2021 ▸). The different possible combinations of correlating a rod with itself, with the neighboring rod, with the next nearest neighbor and with the opposite rod will be represented by *N* = 0, 1, 2, 3, respectively. Therefore, the correlation between different rods results in surfaces in the (*q*_1_, *q*_2_, ϕ) correlation space, which can be parameterized as 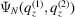 [equation (S4)]. Slices through Ψ shown in Figs. 3 ▸(*e*) and 3 ▸(*f*) are in perfect agreement with the simulated and measured correlation maps. Understanding the correlation maps and the origin of the features can be used to retrieve a more detailed view of reciprocal space. We will first show this for data from numerical calculations, similar to those discussed in Fig. 3 ▸, and afterwards apply the same analysis to the experimental data.

## 4. Analysis of XCCA maps

### 4.1. Numerically generated data

**Figure 4:**
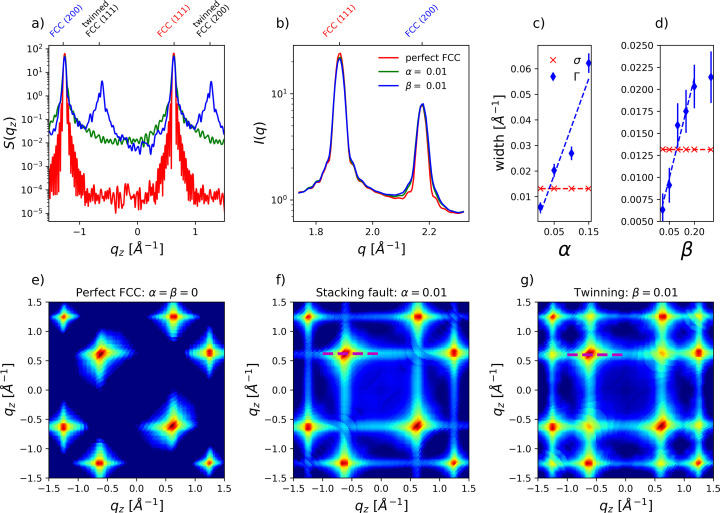
Analysis of data from numerical calculations. (*a*) Averaged *S*(*q_x_*, *q_y_*, *q_z_*) of fault-free FCC crystals (red) and crystals with α = 0.01 stacking fault (green) and β = 0.01 twinning (blue) probability, plotted along a single rod in reciprocal space. (*b*) Azimuthally averaged scattering intensity for the same data as in (*a*). (*c*) and (*d*) Quantitative analysis of the XCCA peak widths, retrieved by Gaussian and Voigt profile fits as detailed in the main text. The error bars are retrieved from the uncertainty of the fits. For σ, error bars are smaller than the symbols. (*e*)–(*g*) 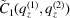 correlation maps for the three cases considered above. The magenta dashed line denotes the location of the FCC (111) self-correlation from neighboring rods, used for the retrieval of Γ.

Fig. 4 ▸(*a*) depicts numerically calculated *S*(*q_z_*) [equation (S2)] for an ensemble of fault-free FCC crystals (red) and for crystals with α = 0.01 stacking fault (green) and β = 0.01 twinning (blue) probabilities. Although *S*(*q_z_*) contains information on the type and density of crystal defects but is not directly accessible in the experiment, the most common observable *I*(*q*) [Fig. 4 ▸(*b*)] lacks a clear distinction between the three considered cases. This is partly due to the fact that only 4 of the 6 FCC (111) lattice points are influenced by stacking faults (*i.e.* are located on rods), which however all overlap in the azimuthal *I*(*q*) average (Warren, 1969 ▸; Velterop *et al.*, 2000 ▸).

A better distinction can be achieved using the corresponding correlation maps 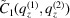, which are displayed in Figs. 4 ▸(*e*)–4 ▸(*g*). In order to reduce the correlation maps to 2D, we introduce 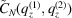, obtained by slicing the 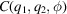 maps along the 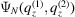 surfaces instead of constant *q*_1_. Therefore, we effectively obtain the correlation along the parabola-like features shown in Figs. 2 ▸ and 3 ▸. For *N* = 1, this representation mostly probes the correlation between two neighboring rods (see also Section 4 of the supporting information), and it contains for a perfect FCC crystal [Fig. 4 ▸(*e*)] the *q_z_* components of the (111) and (200) lattice points. However, additional vertical and horizontal stripes appear for both types of crystal defects as shown in Figs. 4 ▸(*f*) and 4 ▸(*g*). While the number and position of the peaks remain the same for stacking faults (α = 0.01) in Fig. 4 ▸(*f*), twinning defects result in many additional peaks, *e.g.* along the diagonal (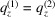) as shown in Fig. 4 ▸(*g*). This can be directly related to the additional peaks also present in *S*(*q_z_*) [Fig. 4 ▸(*a*)], resulting from an inversion of the stacking sequence. However, this level of detail is absent in a data analysis based on *I*(*q*) [Fig. 4 ▸(*b*)], thereby preventing clear identification and distinction between different types of defects.

Not only do the different stacking fault and twinning contributions overlap in the conventional *I*(*q*) representation, but the peak width and position are additionally affected by changes in crystal size and temperature. These influences can be more easily disentangled using XCCA maps. Since the top FCC (111) [red arrow in Fig. 3 ▸(*b*)] is not located on a rod, and the HCP (100) (black arrow) is normal to a rod, both remain unaffected by stacking fault contributions. Therefore, the width at *C*(*q*_1_ = 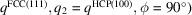 [red dashed line in Fig. 3 ▸(*c*)], fitted with a Gaussian and plotted as red crosses in Figs. 4 ▸(*c*) and 4 ▸(*d*), respectively (σ ∼ 0.0132 Å^−1^), corresponds to the crystal size *d* = 221 Å (Niozu *et al.*, 2021 ▸). The peak position relates to the lattice constant 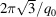 = 5.776 Å, reasonably close to the input values of the simulations (5.779 Å) considering Δ*q* = 0.005 Å^−1^ of the XCCA maps. Importantly, both quantities can be obtained completely unaffected by any influence of stacking faults, which would not be the case for an analysis based on *I*(*q*).

Other features are sensitive to the overall crystal defect density. The main (111) peak [magenta dashed line in Figs. 4 ▸(*f*) and 4 ▸(*g*)] was fit by a Voigt profile, with a Lorentzian width Γ and a Gaussian width 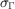. The Gaussian width component, representing the experimental resolution and finite crystal size, was fixed according to the previous results. The Lorentzian widths, Γ, are depicted as blue diamonds in Figs. 4 ▸(*c*) and 4 ▸(*d*). A linear relation is found for both α and β. While this holds until β = 0.2, deviation from a linear relation is observed when the modeled crystallites become more similar to an rHCP structure (α = β = 0.5). Hence, using different features of the correlation maps one can disentangle different properties of the crystallites, such as size, temperature, *i.e.* lattice constant from σ and the density of stacking faults from Γ. Additionally, the presence of crystal twinning manifests itself in the occurrence of additional peaks.

### 4.2. Experimental data

**Figure 5:**
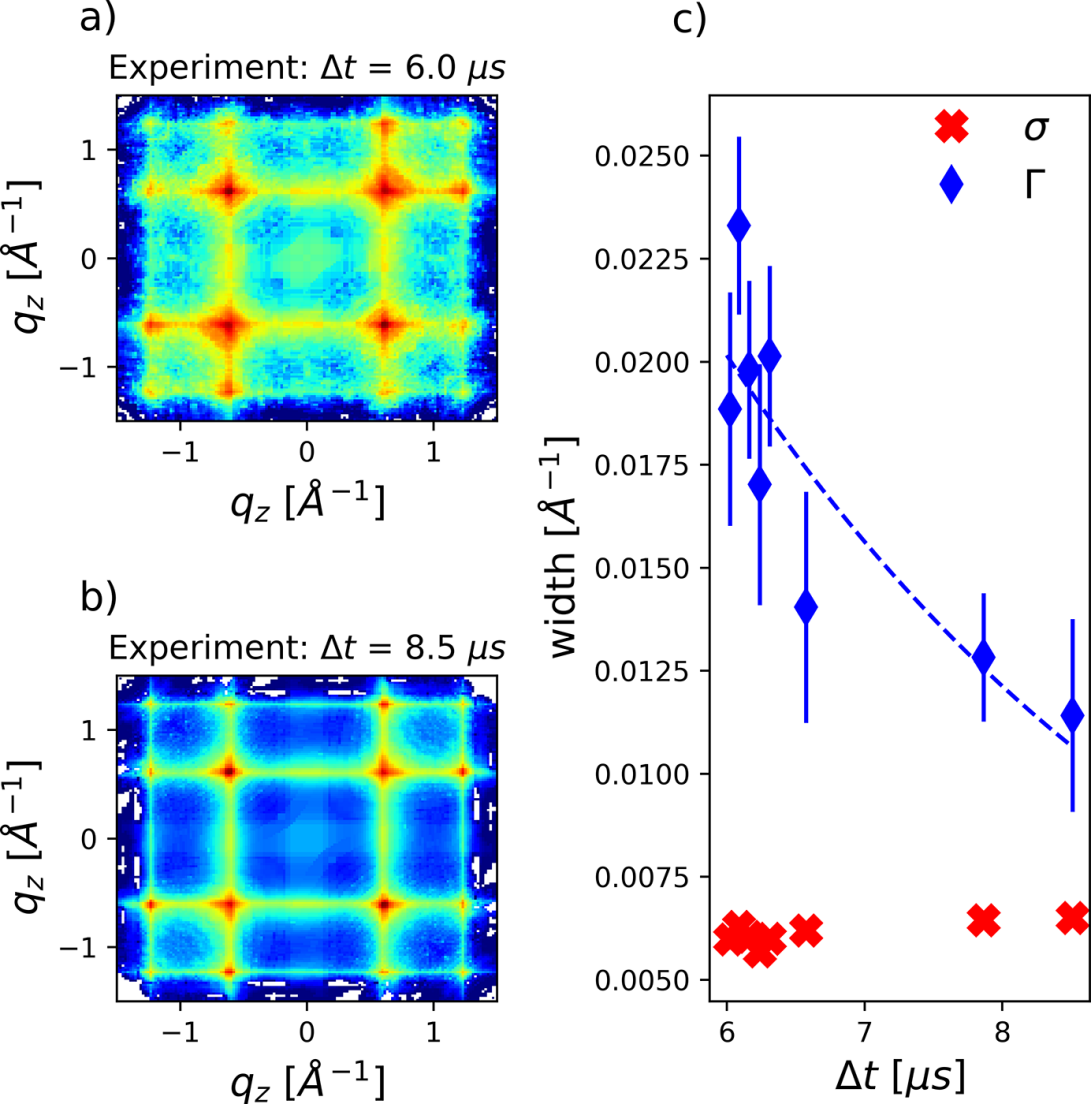
(*a*) and (*b*) Experimental 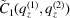 maps for two different times Δ*t* after the onset of cooling. A decrease of stacking fault density becomes apparent due to the evolution of sharper and narrower features in the maps. (*c*) Results of the quantitative peak width analysis of XCCA maps as introduced for simulated data before. While σ can be sensitive to the crystal size, it is diminished by the experimental resolution and therefore constant here. Γ is sensitive to the density of crystal faults. The dashed line is a guide to the eye, illustrating the reduction of stacking fault density with time. Error bars are retrieved from the uncertainty of the fits and are smaller than the symbols for σ.

The same analysis can also be applied to the correlation maps from experimental data, which are displayed for two time points in Figs. 5 ▸(*a*) and 5 ▸(*b*). Overall, the experimental and modeled correlation maps agree qualitatively, confirming the existence of crystalline defects. Distinct peaks along the diagonal (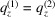) are also present, consistent with significant crystal twinning contributions. Furthermore, the correlation features become sharper with increasing jet propagation time, as also shown by the decreasing width Γ displayed in Fig. 5 ▸(*c*).

Note that, although each probed position along the jet corresponds to a specific propagation time, this does not imply that we are following the time evolution of individual crystallites. Because of the stochastic nature of crystal nucleation, crystals begin forming at random upstream locations and continue to grow as they travel with the jet. Observations at different positions along the jet should therefore not be interpreted as a temporal sequence of crystal growth, but rather as structural characterizations averaged over many statistically independent crystal growth events, each of which originated at different upstream positions and had therefore distinct formation histories.

According to the discussion above, the strong reduction in peak width can unambiguously be assigned to a reduction of crystal fault density within the ensembles. The average crystal size can be obtained unaffected by stacking fault influences from σ [red crosses in Fig. 5 ▸(*c*)], which is found to provide a constant value here. Considering an experimental resolution limited by the bandwidth of the X-rays (Δ*E* ∼ 30 eV), we estimate an additional peak broadening of Δ*q* ∼ 0.006 Å^−1^, closely matching the obtained σ values. Therefore, the measured peak width appears to be mostly determined by the X-ray bandwidth, while additional contributions like pixel size, pixel cross-talk, or instabilities of the setup seem to contribute to a lesser extent. Consequently, only a lower limit for the average crystal sizes *d* > 450 Å can be determined here. This limitation will be overcome in future experiments by the use of narrower bandwidth radiation, *e.g.* by hard X-ray self-seeding (Liu *et al.*, 2023 ▸), which has become available at the MID instrument in the meantime.

The additional peak broadening due to stacking faults, Γ, is well above this resolution limit and therefore shows a strong reduction of crystal fault density. In the jet propagation time window investigated here, between Δ*t* = 6.0 µs and Δ*t* = 8.5 µs, Γ decreases from Γ ∼ 0.02 Å^−1^ to Γ ∼ 0.01 Å^−1^, which is consistent with reductions in β from 0.2 to 0.05 or in α from 0.05 to 0.02–0.03, respectively. Clearly, possible combinations of α, β, or more complex stacking fault morphologies could also result in the same peak width.

## 5. Discussion and outlook

While Ostwald’s rule of stages may provide an explanation for the observed trend in Γ, it is important to recall that our experiment does not track the time evolution of individual crystallites. Other factors may therefore also influence the behavior of Γ. For instance, crystals forming at different positions within the jet may have undergone different thermal histories due to the jet’s temperature profile (Möller *et al.*, 2024 ▸). This variation could affect both the formation and the stability of stacking faults, impacting their prevalence in the averaged correlation maps analyzed here. Because Φ ≥ 0.5 in the region of the jet considered here, crystal nucleation predominantly occurred closer to the nozzle, where the probability of crystal formation is significantly higher (Möller *et al.*, 2024 ▸). Once nucleation takes place, crystals grow rapidly, within a timescale corresponding to only a few micrometres of jet propagation (Schottelius *et al.*, 2020 ▸). As a result, at the propagation times studied in this work, the jet was already mostly crystallized. Still, because of the absence of crystal size information, one cannot distinguish here between a crystal growth process, effectively reducing the density of stacking fault occurrences formed at earlier times, and an annealing process, reducing the absolute number of stacking faults.

Nonetheless, it is evident that a much richer information content can be retrieved from XCCA maps than from *I*(*q*) profiles. The qualitative agreement between the experimental correlation maps and those calculated from a minimal crystal model with defects provides exciting new perspectives for a deeper understanding of structural evolution in the crystallization process.

For example, additional insights into crystal morphology can be obtained from the ratio of the twinned (111) peak intensity to the main (111) peak intensity, which decreases from 0.7 to about 0.5 in our experiment (see Fig. S9). This trend is consistent with a reduction in twinning occurrences. However, these values remain notably higher than those predicted by the simple modeling discussed earlier, suggesting a more complex crystal morphology. We note that a similar level of structural complexity has been observed in the stacking of hexagonal and cubic ice, where stacking probabilities depend on neighboring occurrences (Kuhs *et al.*, 2012 ▸), albeit with much higher overall fault densities.

A particularly promising avenue for future experiments is to probe the liquid jet at distances much closer to the nozzle than those considered in this study. This would enable access to the earliest stages of crystal nucleation and growth, where crystal size plays a crucial role and growth may be strongly suppressed. While the current X-ray bandwidth resolution does not allow direct determination of crystal size, this limitation could be overcome using hard X-ray self-seeding. This advancement should enable detailed investigations into the very first steps of the crystallization process. Given that probing the jet at such early stages would imply working in a region where Φ is reduced by many orders of magnitude, the high repetition rate of the EuXFEL will be essential for obtaining sufficient statistical sampling within a realistic experimental timeframe.

We anticipate that future applications of XCCA will allow for even more complex structural analyses, including higher-order stacking fault descriptions, in-plane lattice distortions, non-hemitropic twinning, or polymorphic occurrences. In this context, a model-free reconstruction of the full 3D reciprocal space could be explored using convolutional neural networks. Notably, the correlation maps 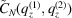 present a promising input for such approaches, as they offer rotational invariance and a significantly reduced data size compared with full 2D detector images while retaining far more structural information than azimuthally integrated *I*(*q*) curves.

In summary, we have shown that femtosecond X-ray scattering in combination with XCCA can be used to study stacking faults in the rapid crystallization of a supercooled atomic liquid. We demonstrated that features such as twinning contributions, which would be hard or impossible to extract from orientational averages *I*(*q*), are preserved, and therefore stacking faults can be detected and distinguished. As not only a few selected hits but the full ensemble is analyzed, the experiment is sensitive to the density of stacking faults, so that a reduction along the jet direction becomes visible. This opens up new experimental opportunities to follow polymorph selection during crystallization, annealing processes in material processing applications, or the structure of metastable ice in atmospherically relevant freezing conditions (Kuhs *et al.*, 2012 ▸; Amaya *et al.*, 2017 ▸; Kim *et al.*, 2023 ▸).

## 6. Related literature

The following references are cited in the supporting information: Hagemann *et al.* (2021 ▸); Kieffer *et al.* (2020 ▸); Stan *et al.* (2016 ▸).

## Supplementary Material

Animated view of the full correlation map. DOI: 10.1107/S2052252525004063/if5006sup1.avi

Supporting figures and equations. DOI: 10.1107/S2052252525004063/if5006sup2.pdf

## Data Availability

Data recorded during the experiment are available at https://in.xfel.eu/metadata/doi/10.22003/XFEL.EU-DATA-002542-00.
