# Supplementary material for: Femtosecond X-ray cross-correlation analysis of disordered crystals forming in a supercooled atomic liquid
Source: IUCrJ. 2025 Jun 3;12(Pt 4):462–71. doi: 10.1107/S2052252525004063 (PMC12224082; doi:10.1107/S2052252525004063)
Supplement: Supplementary file 2 [file m-12-00462-sup2.pdf]

IUCrJ

Volume 12 (2025)

Supporting information for article:

Femtosecond X-ray cross-correlation analysis of disordered crystals forming in a supercooled atomic liquid

Johannes Möller, Michele Caresana, Alexander Schottelius, Felix Lehmkühler, Ulrike Boesenberg, Frédéric Caupin, Francesco Dallari, Tiberio A. Ezquerra, José M. Fernández, Luca Gelisio, Claudia Goy, Jörg Hallmann, Anton Kalinin, Chan Kim, Ruslan P. Kurta, Dmitry Lapkin, Francesco Mambretti, Markus Scholz, Roman Shayduk, René Steinbrügge, Florian Trinter, Ivan A. Vartanyants, Alexey Zozulya, Davide E. Galli, Gerhard Grübel, Anders Madsen and Robert E. Grisenti

SI: Femtosecond X-ray cross-correlation analysis of disordered crystals forming in a supercooled atomic liquid

Johannes Möller,[a*1] Michele Caresana,[b2] Alexander Schottelius,[b] Felix Lehmkühler,[c,d] Ulrike Boesenberg,[a] Frédéric Caupin,[e] Francesco Dallari,[c] Tiberio A. Ezquerra,[f] José M. Fernández,[g] Luca Gelisio,[a] Claudia Goy,[c] Jörg Hallmann,[a] Anton Kalinin,[h] Chan Kim,[a] Ruslan P. Kurta,[a] Dmitry Lapkin,[c3] Francesco Mambretti,[i] Markus Scholz,[a] Roman Shayduk,[a] René Steinbrügge,[c] Florian Trinter,[b,c,j] Ivan A. Vartanyants,[c] Alexey Zozulya,[a] Davide E. Galli,[i] Gerhard Grübel,[c,d4] Anders Madsen[a] and Robert E. Grisenti [b,h*]

[a]*European X-Ray Free-Electron Laser Facility, 22869 Schenefeld, Germany,* [b]*Institut für Kernphysik, J. W. Goethe-Universität Frankfurt am Main, 60438 Frankfurt am Main, Germany,* [c]*Deutsches Elektronen-Synchrotron DESY, 22607 Hamburg, Germany,* [d]*The Hamburg Centre for Ultrafast Imaging, 22761 Hamburg, Germany,* [e]*Institut Lumière Matière, Université Claude Bernard Lyon 1, CNRS, Institut Universitaire de France, 69622 Villeurbanne, France,* [f]*Macromolecular Physics Department, Instituto de Estructura de la Materia, IEM-CSIC, 28006, Madrid, Spain,* [g]*Laboratory of Molecular Fluid Dynamics, Instituto de Estructura de la Materia, IEM-CSIC, 28006, Madrid, Spain,* [h]*GSI Helmholtzzentrum für*

[1] Both authors contributed equally.
[2] Both authors contributed equally.
[3] Current affiliation: Institut für Angewandte Physik, Universität Tübingen, 72076 Tübingen,Germany
[4] Current affiliation: European X-Ray Free-Electron Laser Facility, 22869 Schenefeld, Germany

Schwerionenforschung GmbH, 64291 Darmstadt, Germany, [i]Dipartimento di Fisica, Università degli Studi di Milano, 20133 Milano, Italy, and [j]Molecular Physics, Fritz-Haber-Institut der Max-Planck-Gesellschaft, 14195 Berlin, Germany.

E-mail: johannes.moeller@xfel.eu, grisenti@atom.uni-frankfurt.de

1. Correlation in reciprocal space

A sketch of the scattering geometry in reciprocal space is shown in Fig. S1. The origin (O) and two further points of the reciprocal lattice intersect with the Ewald sphere, depicted in grey. Both reciprocal lattice points are defined by a vector ($\vec{q}_1$ and $\vec{q}_2$) and the angle ϕ between both vectors is given as $\cos(\phi) = \frac{\vec{q}_1 \cdot \vec{q}_2}{q_1 q_2}$. In this specific example, the origin and both lattice points lie in a plane which is orthogonal to the detector plane. In this case, the angle between both points on the detector surface is $\Delta = 180°$, but the actual angle in reciprocal space is smaller due to the curvature of the Ewald sphere. This has the effect that a small part of the correlation map ($\phi < 180°$) cannot be probed, as already earlier pointed out by Mendez et al. (Mendez *et al.*, 2014).

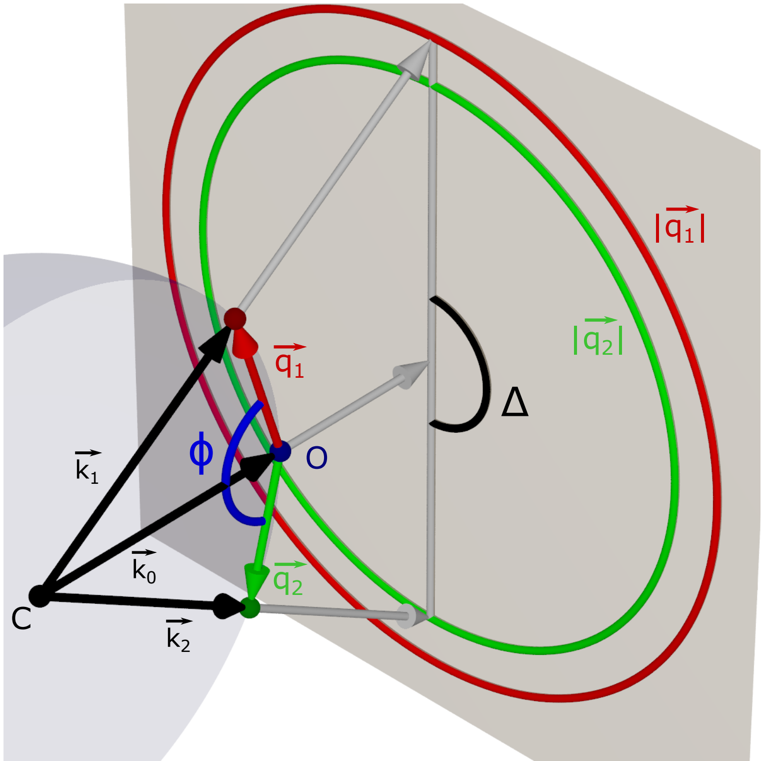

Fig. S1. Sketch of scattering geometry in reciprocal space. Two reciprocal lattice points are displayed, determined by the vectors $\vec{q_1}$ and $\vec{q_2}$. The angle between both is denoted as ϕ in reciprocal space and Δ on the detector surface.

In order to obtain effectively the correlation functions from detector data but directly in reciprocal space, for each pixel of the detector the three-dimensional scattering vector $\vec{q_i} = \{q_i^x, q_i^y, q_i^z\}$ is calculated first (Kieffer *et al.*, 2020). The angle ϕ between two vectors $\vec{q_1}$ and $\vec{q_2}$ is then calculated as the angle between the corresponding pixel vectors. This procedure has also the advantage that only lit pixels (pixels which are above a certain intensity threshold (Möller *et al.*, 2024)) need to be taken into account, as well as the fact that the results are directly obtained in reciprocal space and not on the skewed detector surface. Therefore, this approach doesn't require any regrouping, splitting, or binning of pixels onto a spherical coordinate system, which can introduce artifacts or diminish the angular resolution of the results.

2. Detector corrections

The raw detector data was first treated as described in (Sztuk-Dambietz *et al.*, 2023), identifying gain stages and correcting dark pedestal and slope. However, additional data corrections were observed to be necessary to obtain usable data. Therefore, an additional data correction scheme was developed that removes contributions from background scattering, liquid scattering, and pedestal drifts of the detector. It was already introduced in (Möller *et al.*, 2024) and is summarized again here: Normalization: Each single-shot image is normalized to its low-q scattering intensity to eliminate intensity fluctuations caused by the stochastic nature of SASE radiation. Background Subtraction: Anisotropic scattering backgrounds, primarily from upstream optics, are removed by subtracting the median pixel intensity per pulse train. Liquid scattering is also subtracted, but Bragg reflections are preserved. Pedestal Correction: The median value of each detector module is subtracted to correct for pedestal drifts caused by bias voltage decrease under x-ray illumination. This reduced intensity is denoted $\bar{I}$ and used in the cross-correlation analysis.

3. Calculation of stacking fault structures

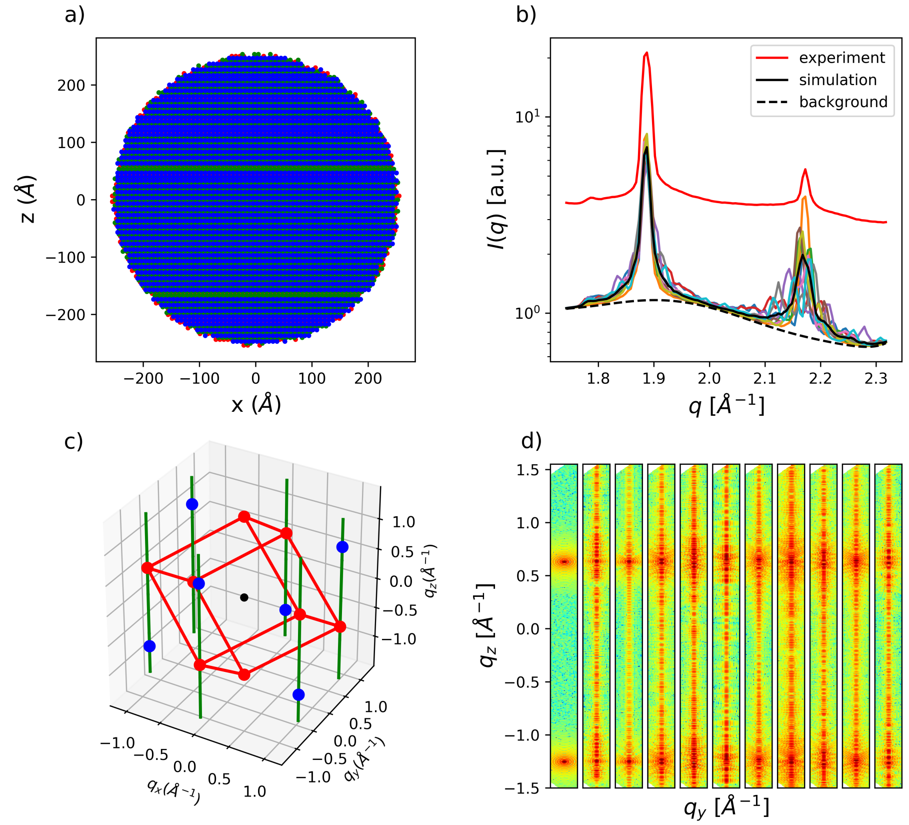

Fig. S2. a) Slice through a 500 Å particle with stacking sequence illustrated as (A) red, (B) green, and (C) blue. Several stacking fault occurrences ($\alpha = 0.05$) can be observed. b) Integration of $S(\vec{q})$ over all directions in reciprocal space for 10 individual particles (500 Å, $\alpha = 0.05$). An instrument background (dashed black line) was added, modelled from experimental data, to achieve a realistic signal-to-background ratio. Also one experimental scattering curve is displayed for comparison (red). c) Three dimensional representation of a reciprocal lattice of an FCC crystal (red: (111), blue: (200)). Additional intensity due to stacking fault imperfections primarily occurs along the vertical rods marked in green. d) Simulated $S(q_x, q_y, q_z)$ plotted along one rod for each of the simulated particles. The left plot shows a perfect FCC crystal (with the same size and shape) for comparison.

A face centered cubic (FCC) crystal structure can be described as a stacked sequence

of close packed atomic planes with six-fold symmetry. Since each layer needs to be shifted laterally to allow for closed packing in three dimensions, either a stacking sequence of ABCABC... for FCC or ABAB... for hexagonal closed packing (HCP) crystal structures is possible. A local deviation from one of these stacking sequences to the other one is one of the most common stacking faults.

We demonstrate the influence of stacking faults by building up crystals with diameters of either 500 Å or 210 Å layer by layer, following the ABC stacking sequence of an FCC crystal. The lattice constant is set to $a = 5.779$ Å of krypton. For each layer, we incorporate a probability of α that the correct next layer is skipped or of β that the sequence is inverted. Following this procedure, we generate $10 - 25$ individual crystals with different randomized stacking sequences for each α and β value. In order to remove any systematic surface scattering effects from the simulations, within a 5 Å thick layer on the particle's surface only 50% of the atom positions are occupied randomly. A slice through one of the generated crystals is displayed in Fig. S2 a).

From these particles, the structure factor in reciprocal space is calculated by Fourier transformation. The reciprocal space was set up as a grid in the range of -2.3 Å$^{-1} \leq q_{x,y,z} \leq 2.3$ Å^{-1}, with a step size of $\Delta q = 0.005$ Å^{-1} resulting in a three-dimensional grid of $921 \cdot 921 \cdot 921$ voxel. Every crystallite's structure factor was calculated on this grid as

$$F(\vec{q}) = \sum_i f(q) \exp(-i \; \vec{q} \; \vec{r_i}), \tag{S1}$$

with i being the index of each atom and $f(q)$ the atomic form factor of krypton.

From this, the modulus squared structure factor (proportional to the scattered intensity) in full reciprocal space follows as

$$S(q_x, q_y, q_z) = F(\vec{q}) \; F^{\star}(\vec{q}). \tag{S2}$$

The spherical average $I(q) = \langle S(\vec{q}) \rangle$ of each particle is shown in Fig. S2 b). The simulated data was scaled for comparable overall intensity and a background, described

by a polynomial (black dashed line), was added. Overall a good agreement between simulations and experiment can be observed. However, the information content of the integrated $I(q)$ curves is limited in contrast to an XCCA map.

A sketch of the reciprocal lattice of a FCC crystal is depicted in Fig. S2 c). The red points show the (111) lattice reflections, which also show the cubic base of the FCC lattice. The blue points depict the (200) reflections. Stacking fault occurrences result in vertical streaks along the q_z direction (green lines), if the stacking direction subject to the faults is z in real space. This is confirmed by slices along the rods displayed in Fig. S2 d) for each of the 10 simulated particles. Additionally, the intensity from a fault-free FCC crystal is shown for comparison. The intensity along the rods contains information about the actual stacking sequence of the particle and is therefore the desired observable for quantifying stacking faults.

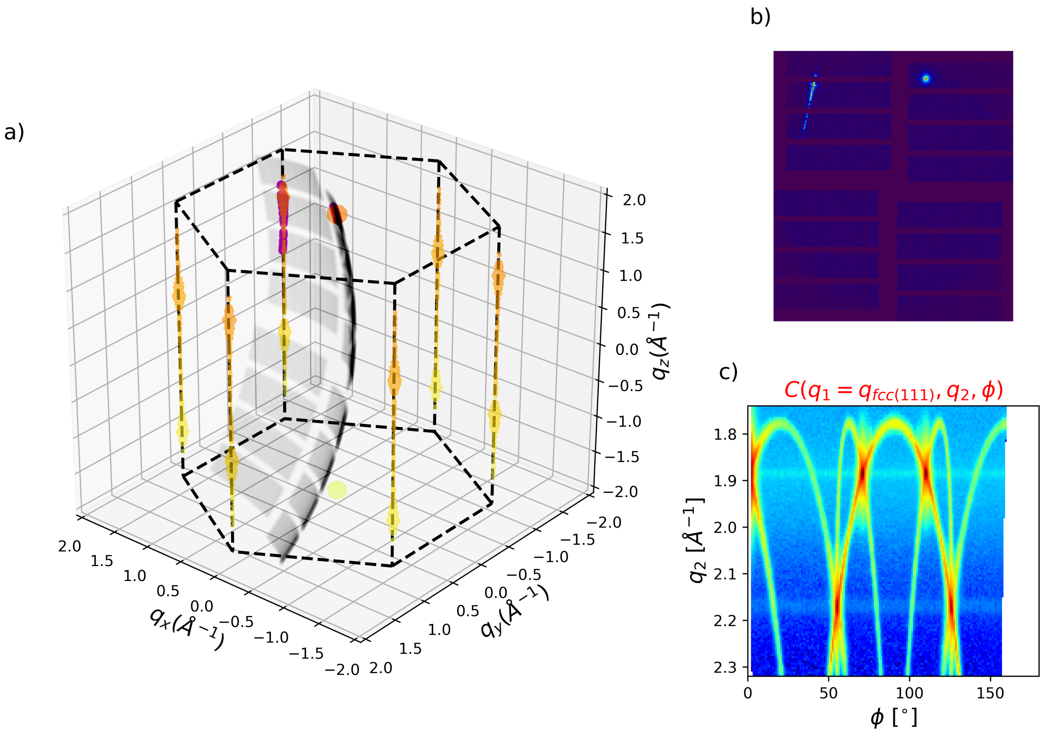

Fig. S3. a) Three-dimensional representation of one simulated particle's $S(q_x, q_y, q_z)$, as also displayed in the main manuscript. b) The two-dimensional detector image as a result of the randomly oriented slice through $S(q_x, q_y, q_z)$. c) Angular cross-correlation map $C(q_1, q_2, \phi)$, shown for $q_1 = q_{FCC(111)}$.

A three-dimensional representation of $S(q_x, q_y, q_z)$ for one simulated particle is shown in Fig. S3 a) and the main manuscript. From the sketched slice, a simulated detector image is generated, by re-scaling, correcting for different angular coverage of different pixels, and adding simulated experimental background, as already shown for the integrated intensity profile in Fig. S2 b). The resulting, single acquisition scattering image on the detector is shown in Fig. S3 b). For each of the ten different particles $75,000$ randomly oriented scattering acquisitions are generated, making overall $N_f = 750,000$ scattering images. Using Eq. 1, their respective correlation map is calculated, without any knowledge of the crystallite orientation in each acquisition.

A slice through the obtained correlation map $C(q_1, q_2, \phi)$ is depicted for one q value corresponding to the (111) peak of FCC, $q_1 = q^{(111)}$, in Fig. S3 c).

4. Geometry in reciprocal space

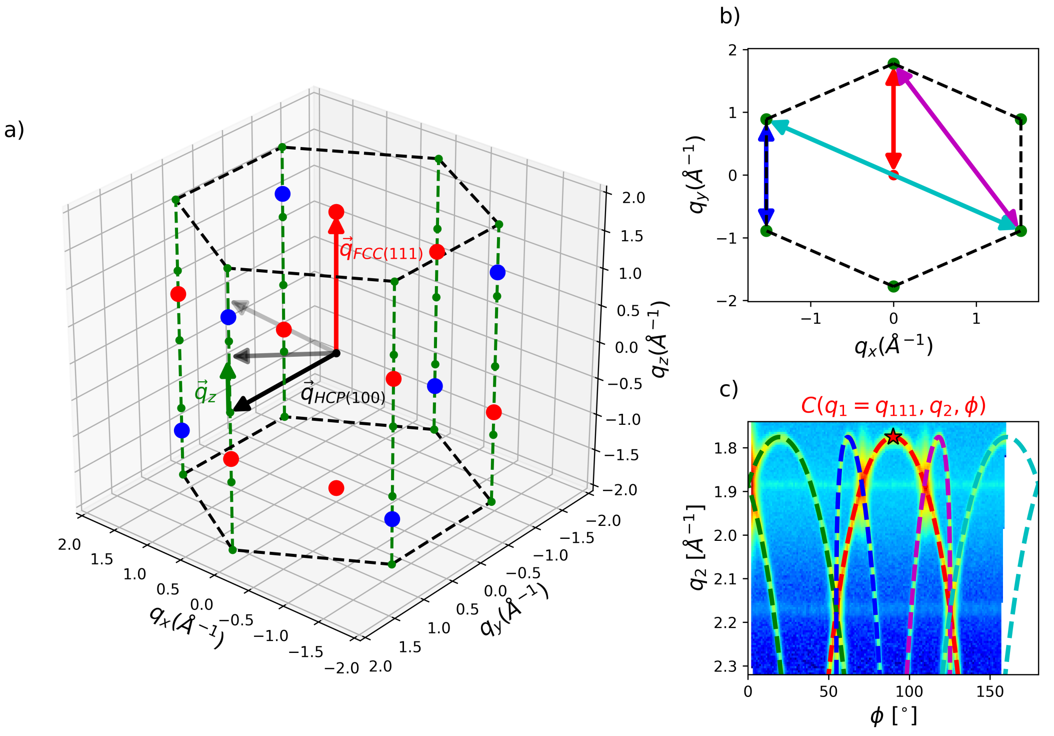

Fig. S4. a) Reciprocal lattice points, as introduced in the main manuscript. b) Top view on the same reciprocal space, showing the different possibilities of correlations between a central (111) lattice point and a rod (red arrow), neighboring rods (blue), next-nearest neighbors (magenta), and oppositely located rods (cyan). c) Calculated lines from geometrical considerations plotted on top of the actual correlation maps. Each line and surface in $C(q_1, q_2, \phi)$ can be attributed to one of the described correlation contributions.

The reciprocal lattice points of FCC and HCP crystals are illustrated in Fig. S4 a). The red vector marks one of these FCC (111) lattice points, while the black arrow marks one of the (100) HCP points. Since the vectors are normal to each other, one would obtain a contribution to the correlation maps at $(q_1 = q^{FCC(111)}, q_2 =$

$q^{HCP(100)}, \phi = 90°)$ if both lattice vectors are present in the reciprocal lattice. This point of the correlation map is marked in Fig. S4 c) by a red star.

Due to the occurrence of stacking faults, many contributions of the structure factor along the green dashed lines occur. Therefore, the central FCC (111) vector (red arrow in Fig. S4 a)) additionally contributes to the correlation maps together with certain (q_2, ϕ) combinations (sketched as black, shaded arrows). We parameterize this by introducing the vector $\vec{q}_z = \vec{q}_2 - \vec{q}_{HCP(100)}$ along the rod [5] (green vector in Fig. S4 a)), so that the contributions in the correlations map occur at (q_1, q_2, ϕ) combinations which follow

$$L(q_z) = \begin{Bmatrix} q_1(q_z) \\ q_2(q_z) \\ \phi(q_z) \end{Bmatrix} = \begin{Bmatrix} q^{(111)} \\ \sqrt{q_z^2 + (q_{HCP})^2} \\ \cos^{-1}\left(\frac{q_z}{\sqrt{(q_{HCP})^2 + (q_z)^2}}\right) \end{Bmatrix}. \tag{S3}$$

This is displayed as a red dashed line in S4 c). However, an inversion, meaning an unambiguous retrieval of the structure factor along a single rod, is not possible from these $C(q_1, q_2, \phi)$ contributions, since neighboring rods have a reversed direction ((111) at positive q_z and (200) at negative q_z, or vice versa) and also the second central FCC (111) at $q_z = -1.88$ Å^{-1} contributes to the same parts of the map. In contrast, this inversion symmetry is not the case if correlations between rods are considered.

Overall, four different rod-rod correlation contributions can be obtained in six-fold symmetry, as also illustrated in Fig. S4 b). Each rod can be correlated with itself (green), a nearest-neighbor rod (dark blue), a second-nearest neighbor (purple), or an oppositely located rod (light blue). If we consider points on the k-th rod $\vec{q}^{(k)} = (q_{HCP}\cos(k\frac{\pi}{3}), q_{HCP}\sin(k\frac{\pi}{3}), q_z)$, we can parameterize the rod-rod correlation surfaces

[5] For simplicity, we will refer to $\vec{q}_{HCP(100)}$ as $\vec{q}_{HCP}$ in the following.

using $(q_z^{(1)}, q_z^{(2)}, N)$, where N stands for the N-th neighboring rod [6]:

$$\Psi_N(q_z^{(1)}, q_z^{(2)}) = \begin{Bmatrix} q_1(q_z^{(1)}, q_z^{(2)}, N) \\ q_2(q_z^{(1)}, q_z^{(2)}, N) \\ \phi(q_z^{(1)}, q_z^{(2)}, N) \end{Bmatrix}$$
$$= \begin{Bmatrix} \sqrt{(q_{HCP})^2 + (q_z^{(1)})^2} \\ \sqrt{(q_{HCP})^2 + (q_z^{(2)})^2} \\ \cos^{-1}\left(\frac{(q_{HCP})^2 \cos(N\frac{\pi}{3}) + q_z^{(1)} q_z^{(2)}}{\sqrt{(q_{HCP})^2 + (q_z^{(1)})^2}\sqrt{(q_{HCP})^2 + (q_z^{(2)})^2}}\right) \end{Bmatrix} \tag{S4}$$

The corresponding contributions in the correlation map are plotted in the same colors on top of a simulated correlation map in Fig. S4 c) as dashed lines.

Slicing the correlations maps $C(q_1, q_2, \phi)$ along the dashed lines $\Psi_N(q_z^{(1)}, q_z^{(2)})$ is introduced as $\tilde{C}_N(q_z^{(1)}, q_z^{(2)})$ in the main manuscript. Although we can define a surface $\Psi_N(q_z^{(1)}, q_z^{(2)})$ for a specific type of rod-rod correlation (e.g. a specific N), the function $\tilde{C}_N(q_z^{(1)}, q_z^{(2)})$ is not uniquely defined in all regions. This is observable in Fig. S4 c) as intersection points. To verify the robustness of our approach, the quantitative information retrieval is demonstrated on simulated data first, which contains the same ambiguity. Therefore, the function $\tilde{C}_N(q_z^{(1)}, q_z^{(2)})$ can still be conveniently used in model-assisted analysis (as demonstrated in this paper), since its behavior at the intersection points can be well reproduced in simulations.

[6] The numerator is the scalar product $\vec{q}^{(1)} \cdot \vec{q}^{(2)}$ and note that $\cos(k\frac{\pi}{3})\cos((k+N)\frac{\pi}{3}) + \sin(k\frac{\pi}{3})\sin((k+N)\frac{\pi}{3})) = \cos(N\frac{\pi}{3})$. In fact the scalar product does not depend on k due to the rod inversion symmetry in the structure factor.

5. Multiple hits and simplification of the calculation

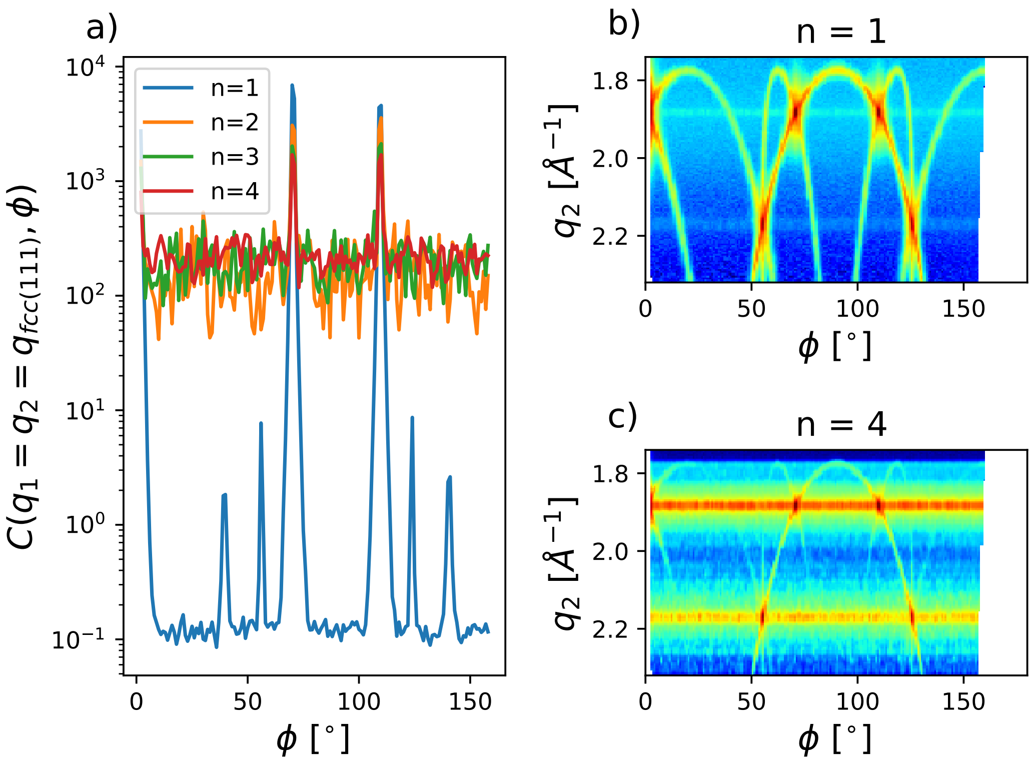

Fig. S5. a) Simulation of XCCA correlation signal $C(q_1 = q_2 = q_{FCC(111)}, \phi)$, with each detector acquisition containing the scattering of n randomly oriented crystals. A clear decrease of the signal-to-background ratio with increasing n is observed. b) and c) $C(q_1 = q_{FCC(111)}, q_2, \phi)$ displayed for $n = 1$ and $n = 4$, respectively.

In order to retrieve quantitative information from the correlation maps, it is necessary to distinguish correlations of a single crystalline structure from any parasitic correlation that can occur during a real experiment, such as crystal-background correlations or correlations between two different crystals. Since the orientation of each crystal is random, such contributions to the correlation maps should not have any angular (ϕ) dependence.

This is investigated by extending the simulation to not only contain one crystal ($n = 1$)

contributions, but also contributions from several crystals in each simulated detector image. The resulting correlation maps are displayed in Fig. S5. While in the case of $n = 1$ only a small ϕ-independent component can be observed at the q values of the (111) and (200) peaks stemming from correlations with the scattering background, these contributions are much more enhanced for $n = 4$. However, as the (q_1, q_2, ϕ) combinations which contribute to certain (skewed) parabolas are known, one can estimate the background from all the other (q_1, q_2, ϕ) combinations which are not on one of the parabolas and subsequently subtract them.

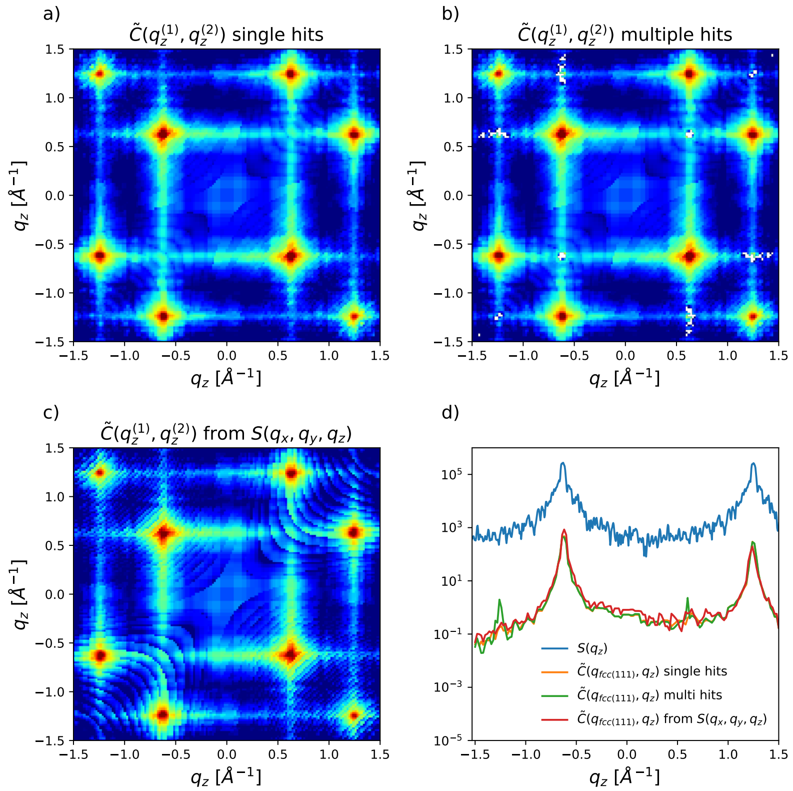

Fig. S6. $\tilde{C}_1(q_z, q_z)$ correlation maps calculated from a) simulated detector images containing single hits, b) a distribution of $n = 1 - 4$ hits, and c) directly in reciprocal space without explicit calculation of detector images. d) Slice through each $\tilde{C}_1(q_z, q_z)$ at the q_z value corresponding to the FCC(111).

To investigate this, an ensemble of detector images is created, which consists equally of single, double, triple, and quadruple hits. After background subtraction and subsequent conversion to $\tilde{C}_1(q_z, q_z)$, the same correlation map can be obtained as from single hit correlations only, as depicted in Fig. S6 a) and b) for single and multiple hits, respectively. A slice through these correlation maps at the q_z value of the (111) reflection is additionally displayed in Fig. S6 d), with orange marking the single-hit and green the multiple-hit simulation. The same correlation function can be obtained, with some additional noise at the intersection points of rods in (q_z, q_z) space. This demonstrates the benefit of using short and tightly focused X-ray pulses for probing these structures, in order to ensure primarily single-hit acquisitions and boost the signal-to-background ratio. Still, even if small fractions of multiple hits remain in the experimental data, they can be removed from the correlation maps.

Additionally, the possibility is demonstrated to calculate $C(q_1, q_2, \phi)$ directly from $S(q_x, q_y, q_z)$ in our simulations, without the need to explicitly simulate many frames on the detector surface. Therefore, as compared to Eq. (1) in the main manuscript, the summation over the frames can be removed, probing all possible orientations directly at once as

$$C(q_1, q_2, \phi) = \frac{1}{N_\Delta(q_1, q_2, \phi)} \sum_{\vec{q}_j \in H(q_1)} \sum_{\vec{q}_k \in H(q_2)} S(\vec{q}_j)\ S(\vec{q}_k)\ \delta(\phi, \vec{q}_j, \vec{q}_k). \tag{S5}$$

$$H(q) = \{\vec{q}_i \mid S(\vec{q}_i) > 10\ arb.u. \text{ and } |\vec{q}_i| = q \pm \Delta q\}. \tag{S6}$$

$$N_\Delta(q_1, q_2, \phi) = 2 * \pi / N(\phi) * N(q_1) * N(q_2) * \sin(\phi), \tag{S7}$$

with $N(\phi)$ being the number of discrete ϕ steps and $N(q_i)$ the number of points of the reciprocal space grid which are within $|\vec{q}_i| = q \pm \Delta q/2$. After averaging over the 10 different crystal versions, the resulting correlations are shown in Fig. S6 c) and as red line in d). This demonstrates that correlations directly from a simulated $S(q_x, q_y, q_z)$ are equivalent to the correlation from the simulated scattering images containing realistic

scattering geometry, number of frames, background, and intensity, which simplifies the calculations in the following significantly.

For comparison, the actual $S(q_z)$, which served as an input to the numerical calculations but is not accessible directly in an experiment, is displayed in Fig. S6 d) as well. With this, one can see that indeed the structure factor along rods formed in reciprocal space can be probed.

6. Influence of burst-mode acquisition scheme

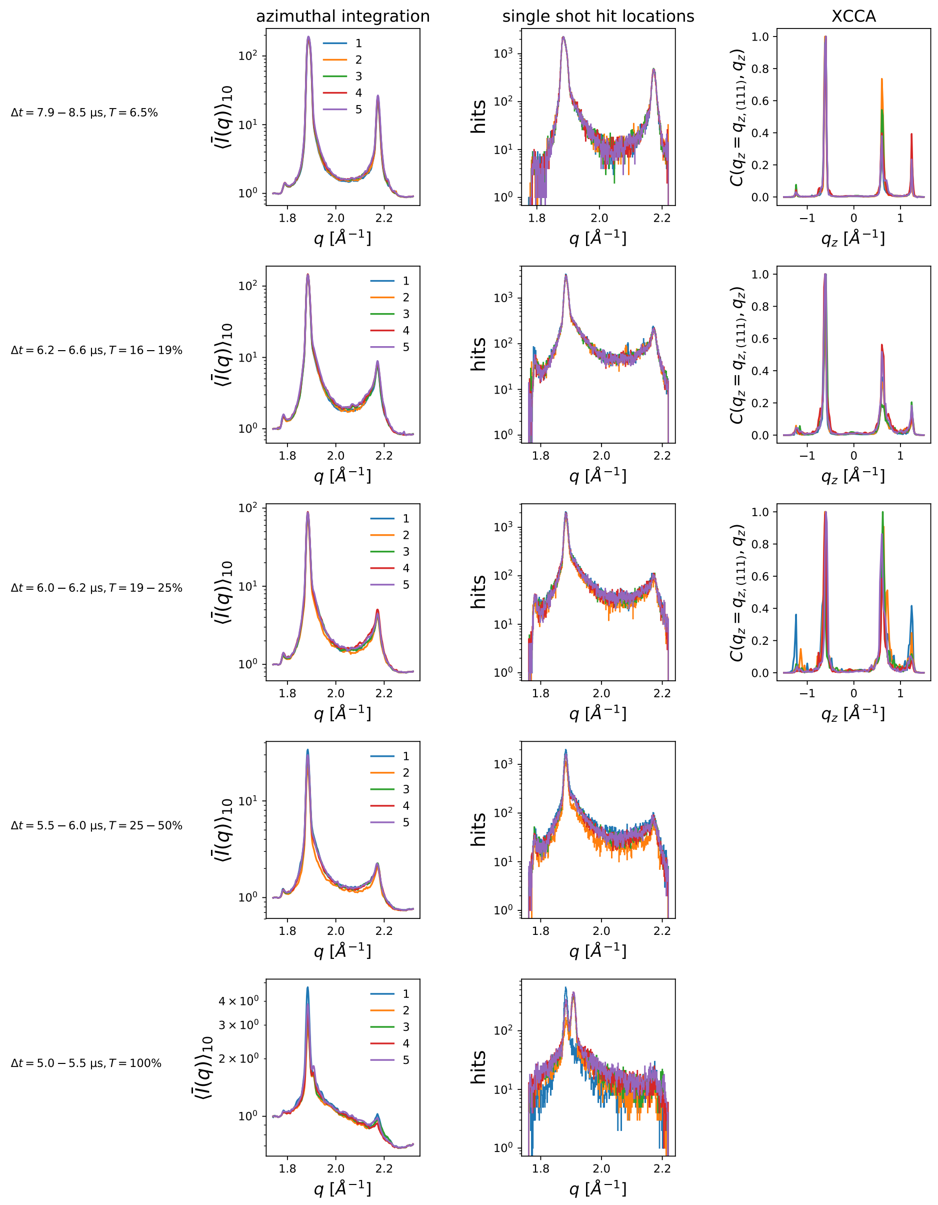

Fig. S7. Influences of the burst-mode acquisition scheme on the measured data. Each plot contains data separately calculated for the first, second, third, fourth, and fifth pulse of each train. The first column compares the azimuthally integrated intensity of each pulse number, the second column the location of detected hits on the detector surface, and the third column the XCCA signal at the most intense FCC (111) peak. Due to the reduction of crystal hits at smaller Δt, no XCCA maps could be calculated for the runs at $\Delta t <= 6.0$ μs.

The European XFEL delivers X-rays in short bursts (trains), with an inter-train repetition rate of 10 Hz. The intra-train repetition rate was set in the presented experiment to 1.1 MHz. While the fast speed of the jet is typically enough to replenish the probed volume after an X-ray illumination within < 880 ns, the subsequent jet rupture can create shockwaves traveling along the jet (Stan *et al.*, 2016; Hagemann *et al.*, 2021).

Fig. S7 displays the reduced intensity (first column), histogram of peak positions (second column), and XCCA signal (third column) for different regimes of our measurement campaign, each plotted separately for the first, second, third, fourth, and fifth pulse of the trains. While at large distances from the nozzle ($\Delta t > 7.9$ μs, first row) a fully crystallized jet is probed, the measurements were performed with reduced X-ray intensity (transmission $T = 6.5\%$) in order to avoid over-illumination of the detector. In this regime, no difference between the first or any subsequent pulse (different colors in the plot) can be observed. This statement is also true for the measurements displayed in the second and third row, for which incrementally a higher transmission was used. In the fourth and fifth row however, in which measurements with up to 100% transmission are displayed, noticeable differences can be observed between the first and any other pulse of a train. Therefore, we exclude those measurements from further analysis and include only data within the range $\Delta t = 6.0$ μs - $\Delta t = 8.5$ μs.

7. Synchrotron measurements

Supporting measurements were performed at beamline P01 of PETRA III, DESY (Wille *et al.*, 2010) in 40-bunch mode (192 ns bunch separation). The beam was focused to a spot size of about 1.2 μm x 6.6 μm. A krypton jet of 10 μm thickness was used. Scattering data was recorded with a Pilatus 1M detector, whose acquisition time was adapted to ensure single-pulse exposure.

A sum image of $30,000$ single acquisitions is displayed in Fig. S8 a), and a single acquisition in b). Blue-shaded regions are the locations of the (111) and (200) reflections. While the same signature of a FCC structure can be obtained in the summed (a) and azimuthally integrated image (c), the single acquisitions contain only sparse, single-photon data from which no streak appearance of the peaks can be identified. Still, an XCCA signal can be calculated from this sparse data set. Due to the reduced scattering intensity, however, only at the location of the most intense reflections, but not the full 3D correlation map as in Fig. 2. The obtained correlations are displayed in d) and e). The red and black lines in d) correspond to the dashed lines shown in the main manuscript Figs. 2 b) and c). The same features from FCC (peaks at $\phi = 70.5°$ and $\phi = 109.5°$ for $q_1 = q_2 = q^{(111)}$ and $\phi = 90°$ for $q_1 = q_2 = q^{(200)}$) and stacking faults (additional peaks) can be observed, as described in the main text. This further confirms the results obtained by our XFEL study, but also demonstrates possible applications for XCCA in synchrotron studies in the future, paving the way to ultra-fast, single-shot synchrotron diffraction experiments. Investigations of the full q-range and on only partly crystallized samples are only possible using the higher flux of XFEL sources, however.

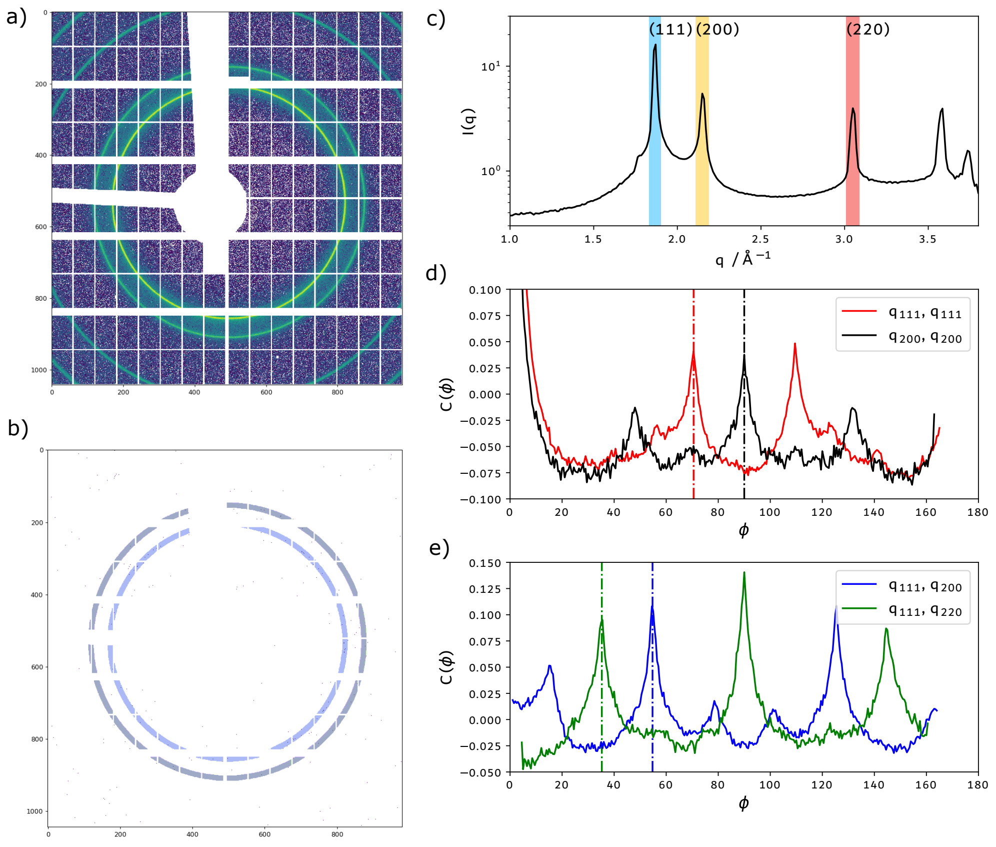

Fig. S8. a) Summed scattering intensity of 30, 000 acquisitions at beamline P01. b) A representative single acquisition. Blue shaded areas are the locations of (111) and (200) reflections of FCC. Occasional blue dots are single photon occurrences. c) Azimuthal integration of the data displayed in a). d) XCCA signal calculated for the (111) and (200) lattice vectors, equivalent to the black dashed line in Fig. 2 a) and b) of the main manuscript. Dashed dotted lines mark the single crystal FCC peaks, while additional peaks due to stacking faults can be found. e) XCCA signal for the (111) vector correlated with the (200) and (220) vectors.)

8. Comparison

Fig. S9 displays slices of the correlation maps obtained from the experimental data a) and from modelled structures containing stacking faults b) and twinning c). The locations of the FCC (111) and (200) peaks are marked by black, dashed lines. In the case of twinning, additional peaks become visible, which can be rationalized by

the inverted stacking direction resulting in an inversion of q_z in reciprocal space. This feature is clearly visible in the experimental data as well. It is worth noting that the experimental data features weaker (200) and stronger twinned (111) peaks as compared to the simulated twinning data. This hints to an even higher information content within the XCCA maps, which could be harvested in future studies with more complex modeling as layed out in the main manuscript.

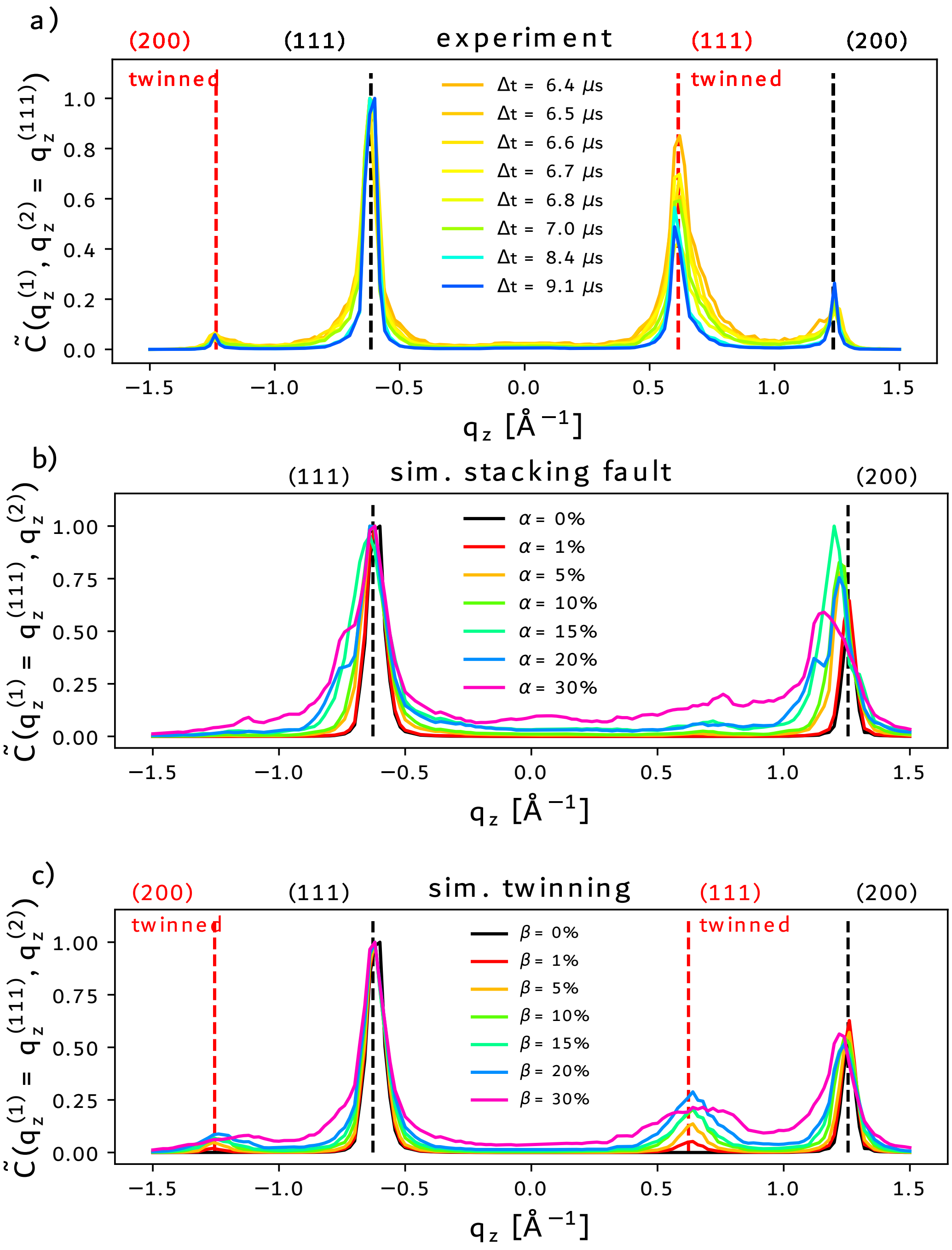

Fig. S9. a) Slice through the correlation maps obtained from experimental data, measured at different times after the onset of cooling. The location of the FCC (111) and (200) peaks are marked by black, dashed line. Contributions from twinned FCC are marked by red lines. b) Slices through correlation maps from simulated data for different stacking fault probabilities α. c) Slices through correlation maps from simulated data for different twinning probabilities β.

References

Hagemann, J., Vassholz, M., Hoeppe, H., Osterhoff, M., Rosselló, J. M., Mettin, R., Seiboth, F., Schropp, A., Möller, J., Hallmann, J. *et al.* (2021). *Journal of Synchrotron Radiation*, **28**(1), 52–63.

Kieffer, J., Valls, V., Blanc, N. & Hennig, C. (2020). *Journal of Synchrotron Radiation*, **27**(2), 558–566.

Mendez, D., Lane, T. J., Sung, J., Sellberg, J., Levard, C., Watkins, H., Cohen, A. E., Soltis, M., Sutton, S., Spudich, J. *et al.* (2014). *Philosophical Transactions of the Royal Society B: Biological Sciences*, **369**(1647), 20130315.

Möller, J., Schottelius, A., Caresana, M., Boesenberg, U., Kim, C., Dallari, F., Ezquerra, T. A., Fernández, J. M., Gelisio, L., Glaesener, A. *et al.* (2024). *Physical Review Letters*, **132**(20), 206102.

Stan, C. A., Milathianaki, D., Laksmono, H., Sierra, R. G., McQueen, T. A., Messerschmidt, M., Williams, G. J., Koglin, J. E., Lane, T. J., Hayes, M. J., Guillet, S. A. H., Liang, M., Aquila, A. L., Willmott, P. R., Robinson, J. S., Gumerlock, K. L., Botha, S., Nass, K., Schlichting, I., Shoeman, R. L., Stone, H. A. & Boutet, S. (2016). *Nature Physics*, **12**, 966.

Sztuk-Dambietz, J., Klujev, A., Laurus, T., Trunk, U., Ahmed, K., Meyer, O., Möller, J., Parenti, A., Raab, N., Shayduk, R. *et al.* (2023). *Frontiers in Physics*, **11**, 1329378.

Wille, H. C., Franz, H., Röhlsberger, R., Caliebe, W. A. & Dill, F. (2010). *Journal of Physics: Conference Series*, **217**(1), 012008.

iucr
